# Supplementary material for: Constructing flexible sub-nanometer ferroelectric catalyst to overcome heterocatalytic kinetic barriers for enhanced catalytic and immuno-therapy
Source: Nat Commun. 2025 Dec 16;17:404. doi: 10.1038/s41467-025-67097-6 (PMC12796250; doi:10.1038/s41467-025-67097-6)
Supplement: Supplementary file 1 — Supplementary Information [file 41467_2025_67097_MOESM1_ESM.pdf]

Supplementary Information

Constructing flexible sub-nanometer ferroelectric catalyst to overcome heterocatalytic kinetic barriers for enhanced catalytic and immuno-therapy

Rui Zhang,[1] Yuanfei Yao,[2] Lu Yang,[1,*] Xudong Zhao,[1] Boshi Tian,[1] Dan Yang,[1,*] and Piaoping Yang[1,*]

[1]Key Laboratory of Superlight Materials and Surface Technology, Ministry of Education, College of Materials Science and Chemical Engineering, Harbin Engineering University, Harbin 150001, P. R. China.

[2]Department of Gastrointestinal Medical Oncology, Harbin Medical University Cancer Hospital, Harbin 150000, P. R. China.

Correspondence and requests for materials should be addressed to L. Y. (email: yanglu@hrbeu.edu.cn), D. Y. (email: yangdan@hrbeu.edu.cn), or P. Y. (email: yangpiaoping@hrbeu.edu.cn).

The supplementary information includes:

1. Supplementary Figures (Fig. 1-41)
2. Supplementary Discussion
3. Supplementary References

1. Supplementary Figures

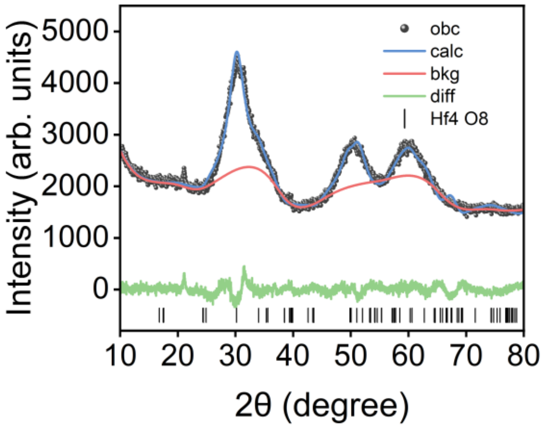

Supplementary Fig. 1. XRD patterns and the corresponding Rietveld refinement of HZO NWs.

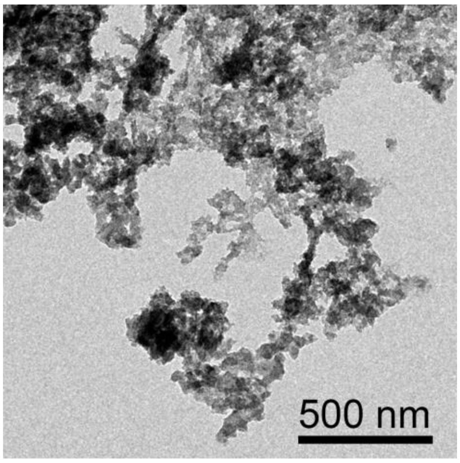

Supplementary Fig. 2. TEM image of HZO NPs.

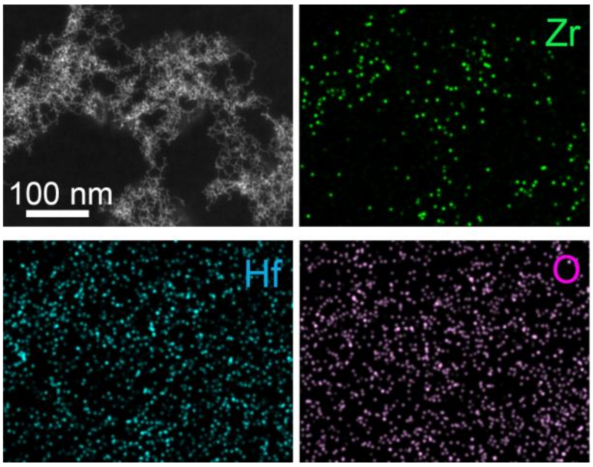

Supplementary Fig. 3. Elemental distribution mapping of the HZO NWs.

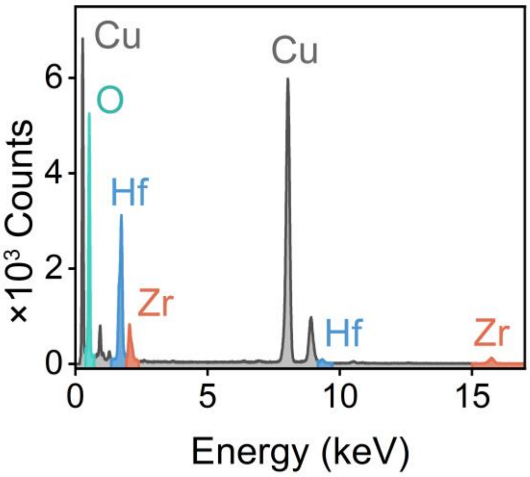

Supplementary Fig. 4. Energy dispersive survey spectrum (EDS) of HZO NWs.

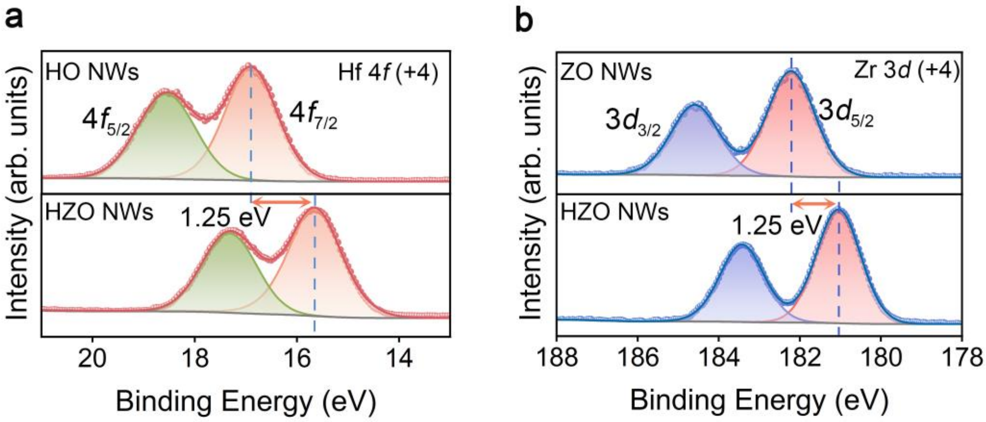

Supplementary Fig. 5. a Hf 4*f* and **b** Zr 3*d* high-resolution XPS spectra of HO NWs, ZO NWs, and HZO NWs.

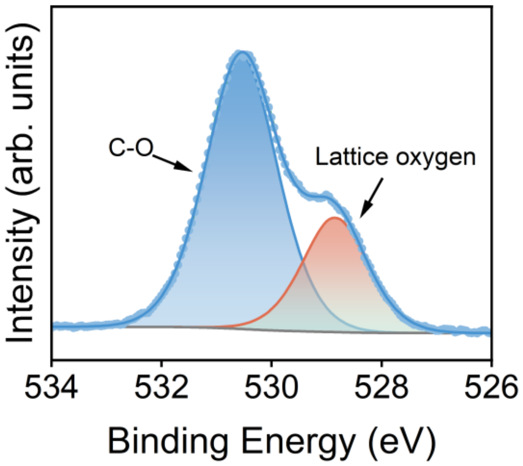

Supplementary Fig. 6. O 1*s* high-resolution XPS spectra of HZO NWs.

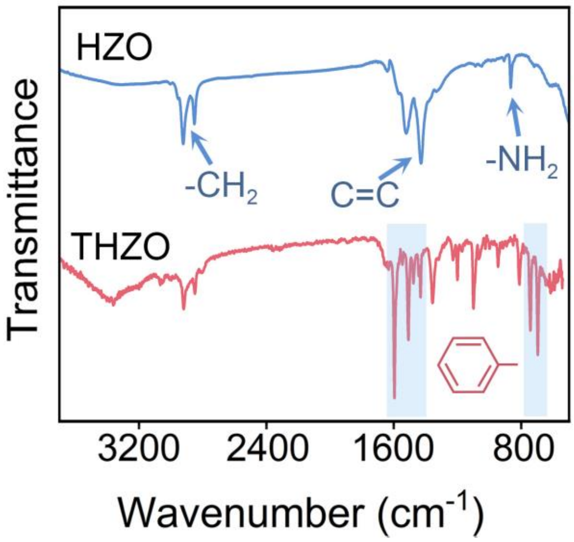

Supplementary Fig. 7. FT-IR spectra of HZO NWs and THZO NWs.

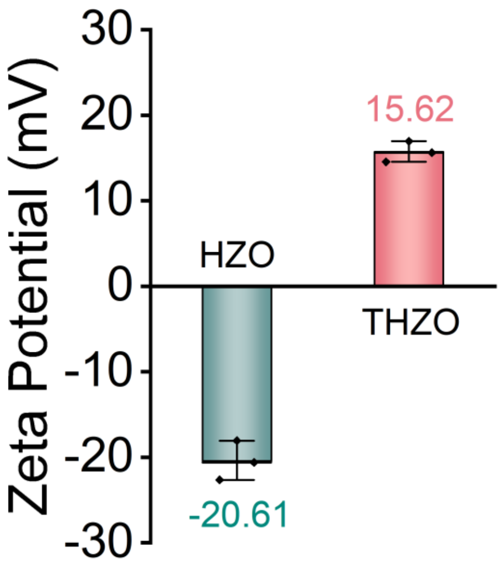

Supplementary Fig. 8. Zeta potential values of HZO NWs and THZO NWs. Data are represented as mean ± S.D. ($n = 3$ represents three independent samples).

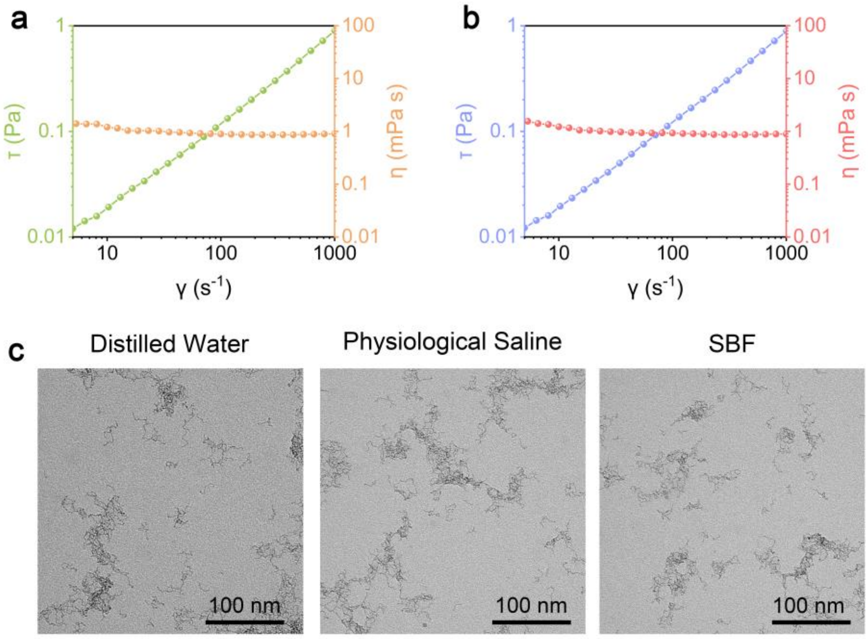

Supplementary Fig. 9. The test curves of kinetic viscosity of THZO NWs in **a** physiological saline and **b** physiological saline solutions containing SBF. **c** TEM images of THZO NWs in different environments.

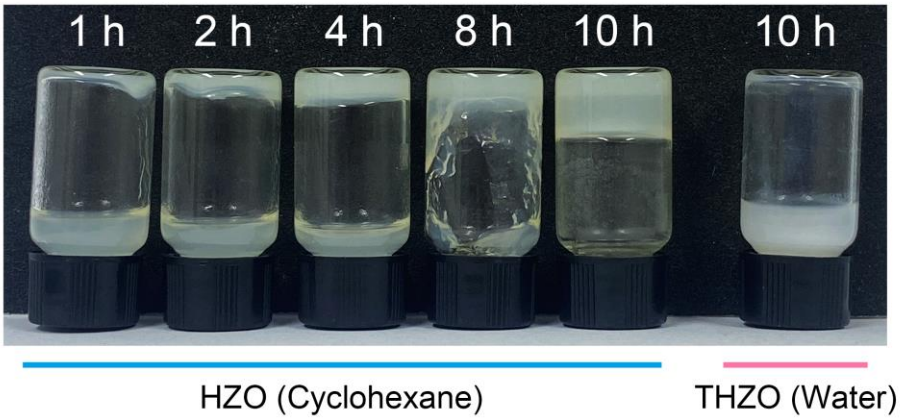

Supplementary Fig. 10. The digital photographs of the gels formed by HZO NWs dispersed in cyclohexane and a digital photograph of THZO NWs in aqueous solution after modifying TPP.

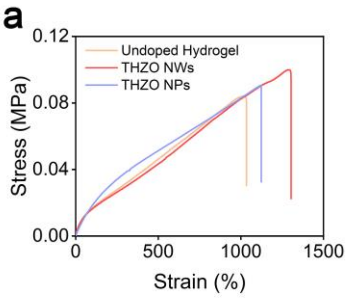

b

	Toughness (MJ m^{-3})	Modulus (KPa)
Undoped Hydrogel	0.49	27.91
HZO NPs	0.60	22.72
HZO NWs	0.72	38.29

Supplementary Fig. 11. a Tensile stress-strain curves of undoped hydrogels, containing THZO NPs and THZO NWs. **b** Young's modulus and toughness.

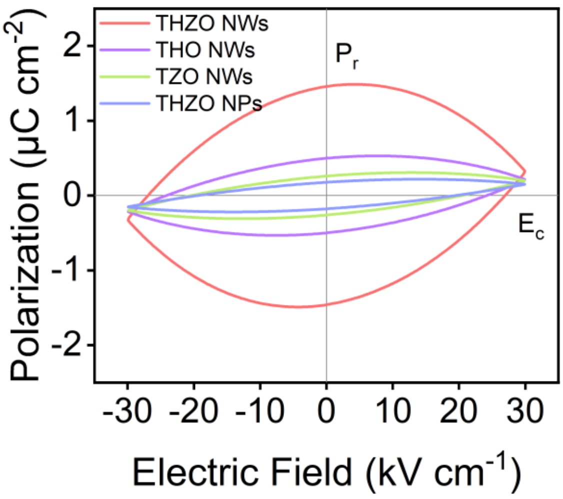

Supplementary Fig. 12. Ferroelectric hysteresis loop of THZO NWs, THO NWs, TZO NWs, and THZO NPs samples at 30 kV voltage.

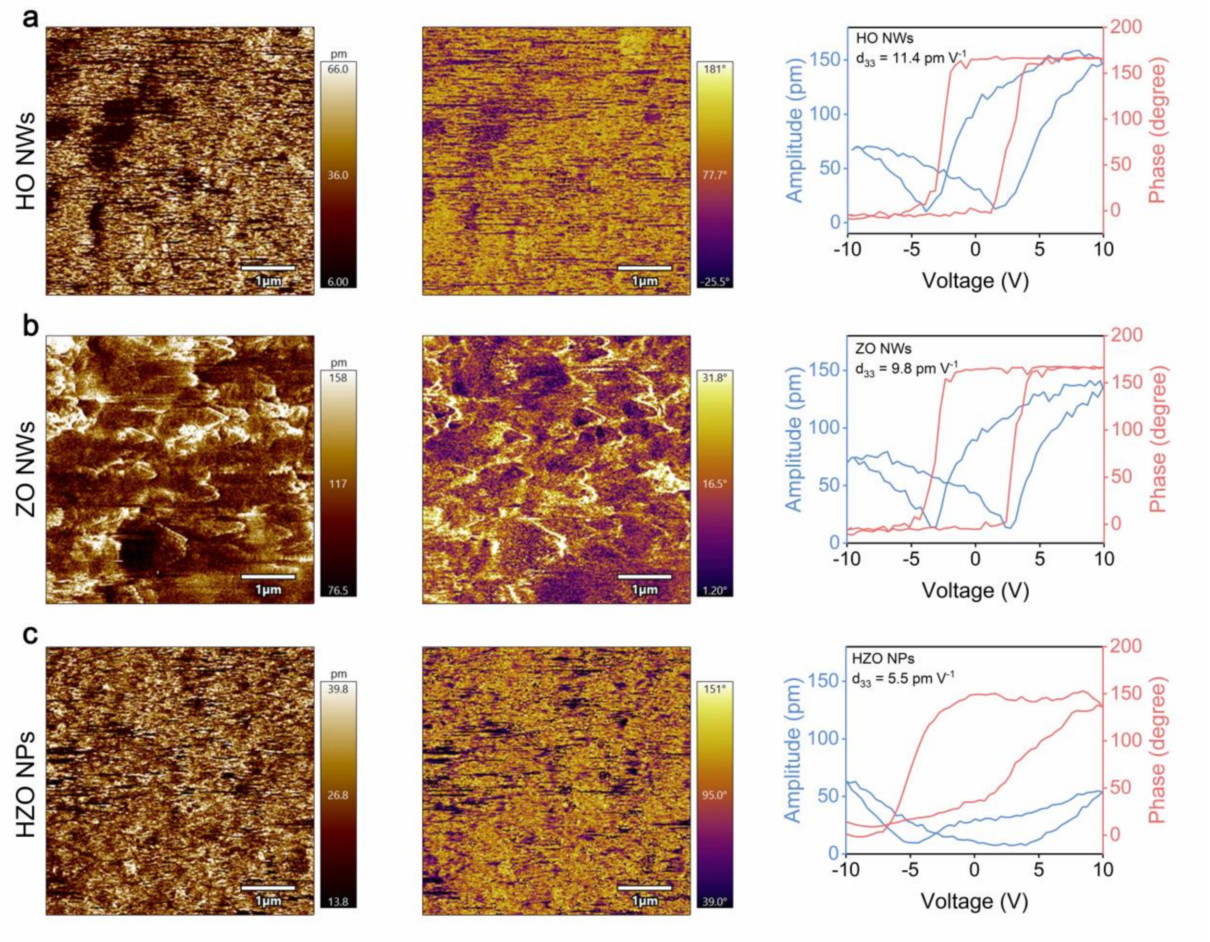

Supplementary Fig. 13. PFM characterization of **a** HO NWs, **b** ZO NWs, and **c** HZO NPs showing amplitude images, phase images and corresponding phase and displacement response curves.

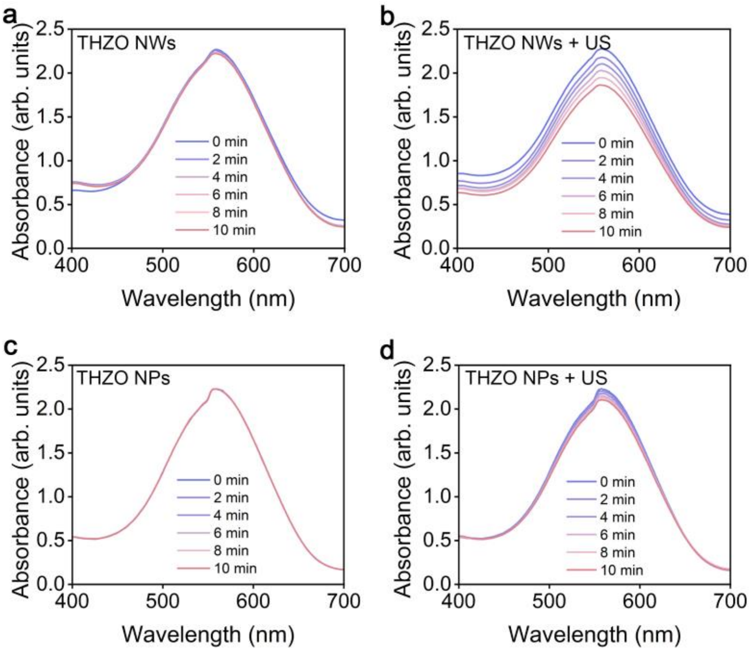

Supplementary Fig. 14. Relationship between UV-*vis* absorption peak intensity of PTIO as a function of irradiation time under the different treatment conditions of **a** THZO NWs, **b** THZO NWs + US, **c** THZO NPs, and **d** THZO NPs + US.

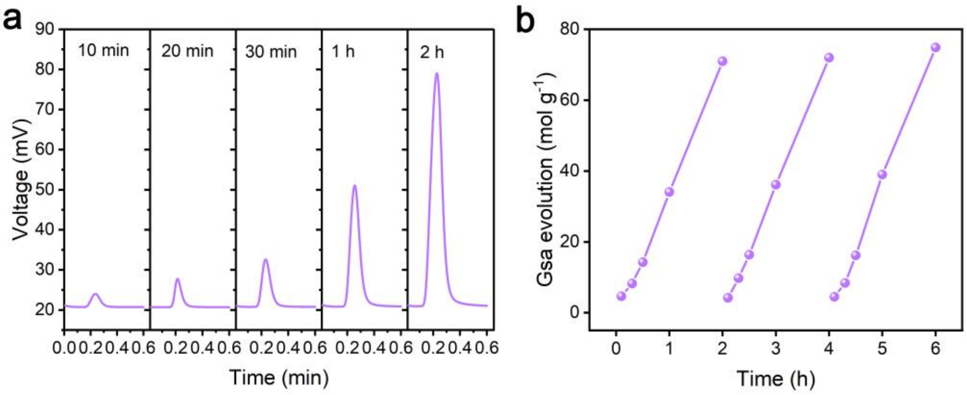

Supplementary Fig. 15. a GC analysis of generation hydrogen. The peak at 0.22 min is the hydrogen signal. **b** The amount of recirculated H_2 precipitation per gram of THZO SNWs under 40 kHz ultrasonic vibration.

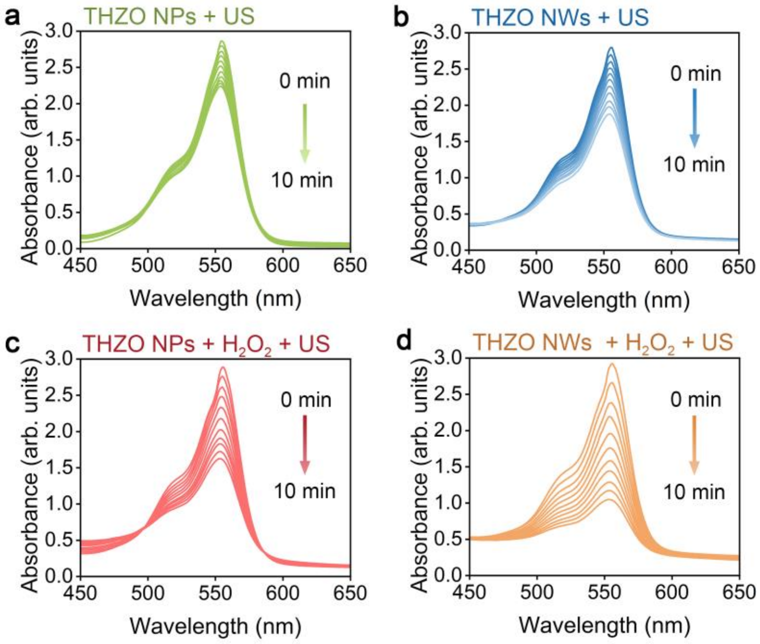

Supplementary Fig. 16. The effects of THZO NPs + US, THZO NWs + US, THZO NPs + H_2O_2 + US and THZO NWs + H_2O_2 + US on the degradation of RhB reaction.

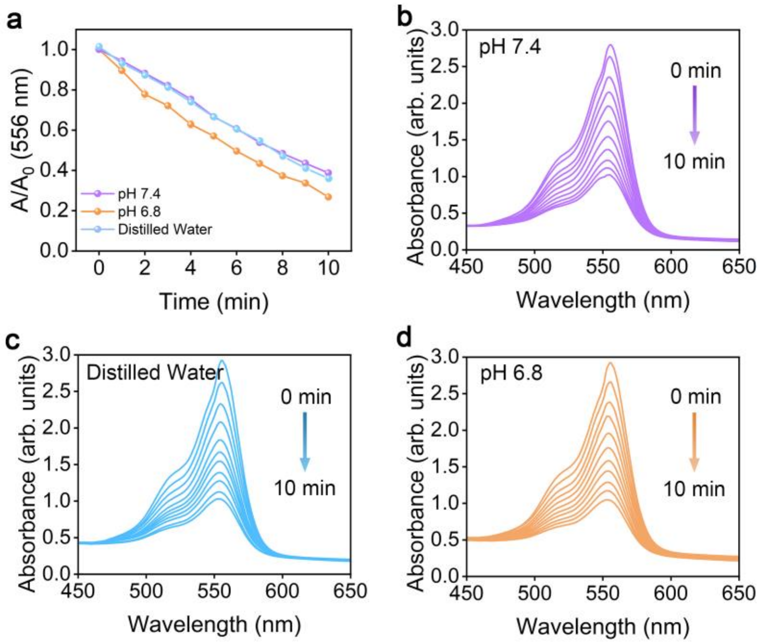

Supplementary Fig. 17. a The effects of THZO NWs + H_2O_2 + US on the degradation of RhB reaction in different solution environments. UV-*vis* absorption peak spectra of RhB in **b** pH=7.4 buffer solution **c** distilled water **d** pH=6.8 buffer solution as a function of US irradiation time.

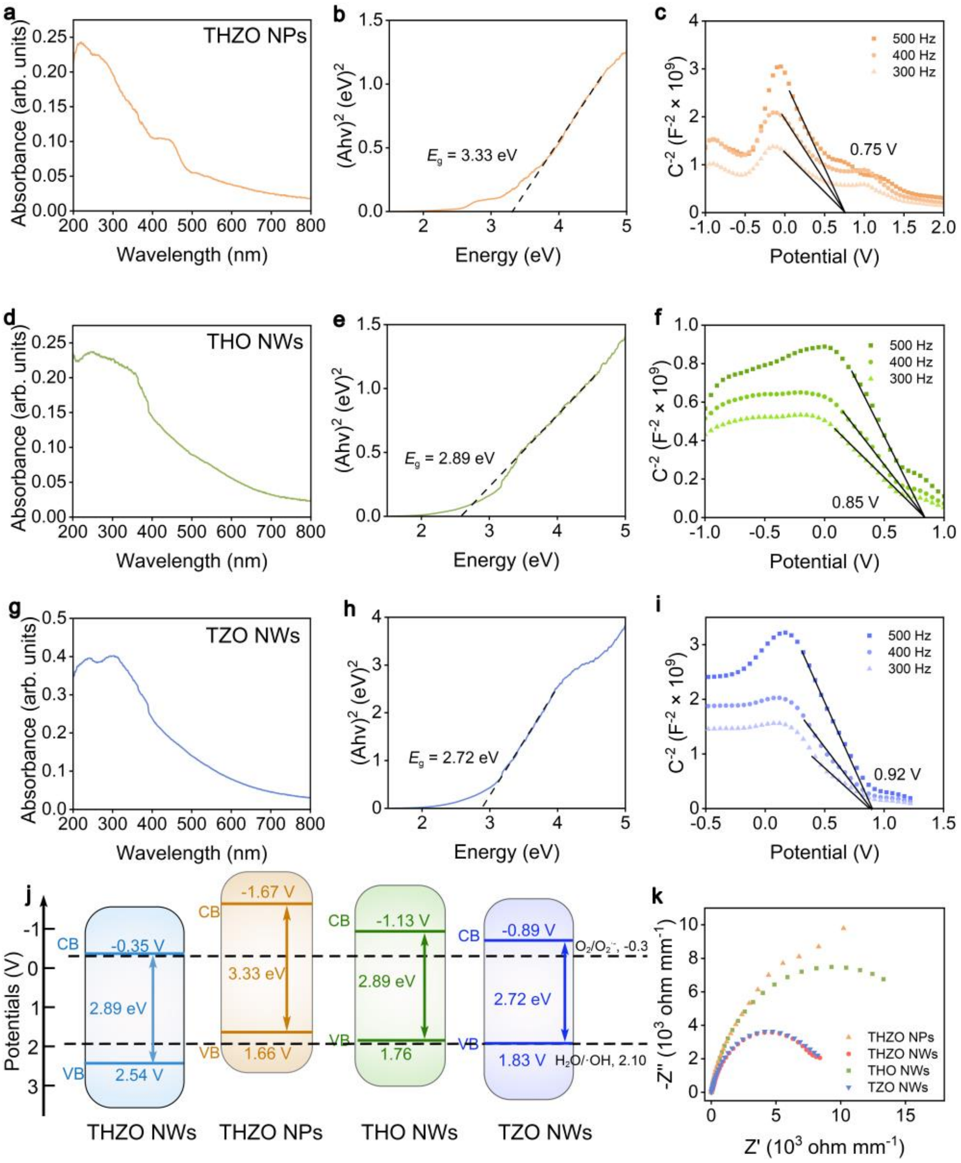

Supplementary Fig. 18. The UV-*vis* diffuse reflectance spectra (**a, d, g**). The bandgap determined using Kubelka–Munk equation (**b, e, h**). The Mott Schottky (M-S) diagrams with different frequency (**c, f, i**) **j** Band structure diagram of THZO NWs, THZO NPs, THO NWs, and TZO NWs. **k** EIS spectra of THZO NWs, THZO NPs, THO NWs, and TZO NWs.

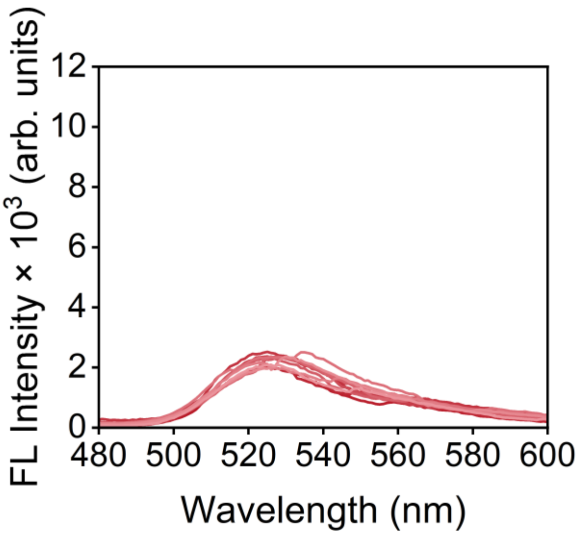

Supplementary Fig. 19. Relationship between THZO NWs and DHR123 with time.

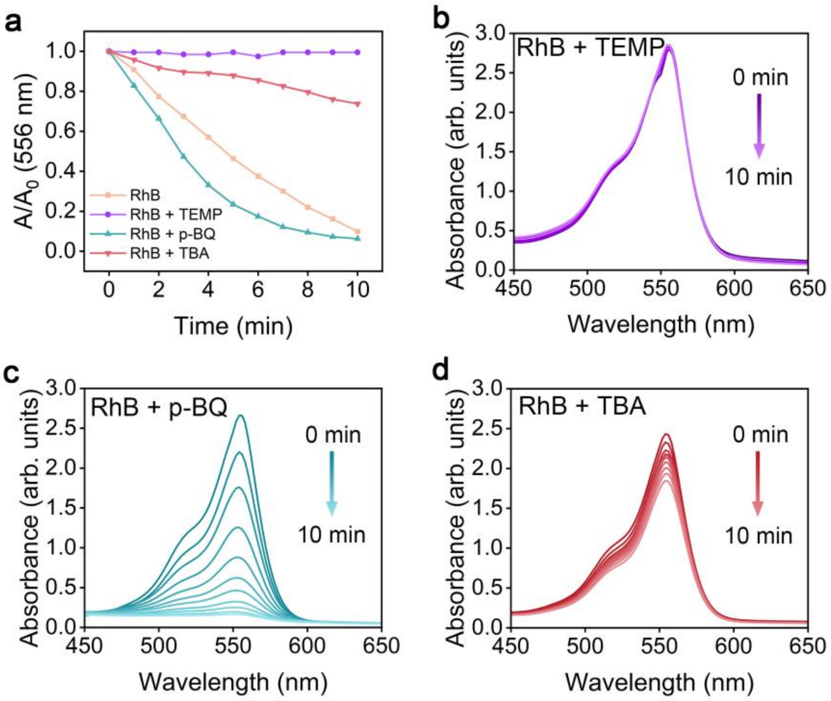

Supplementary Fig. 20. a Degradation experiment of RhB with time under different sacrificial agents. **b** Degradation spectras of the sacrificial agent p-BQ ($O_2^{\cdot-}$). **c** The degradation spectrums of the sacrifice agent TBA (·OH) was added. **d** Degradation spectra of sacrificial agent TEMP for $^1O_2$ added.

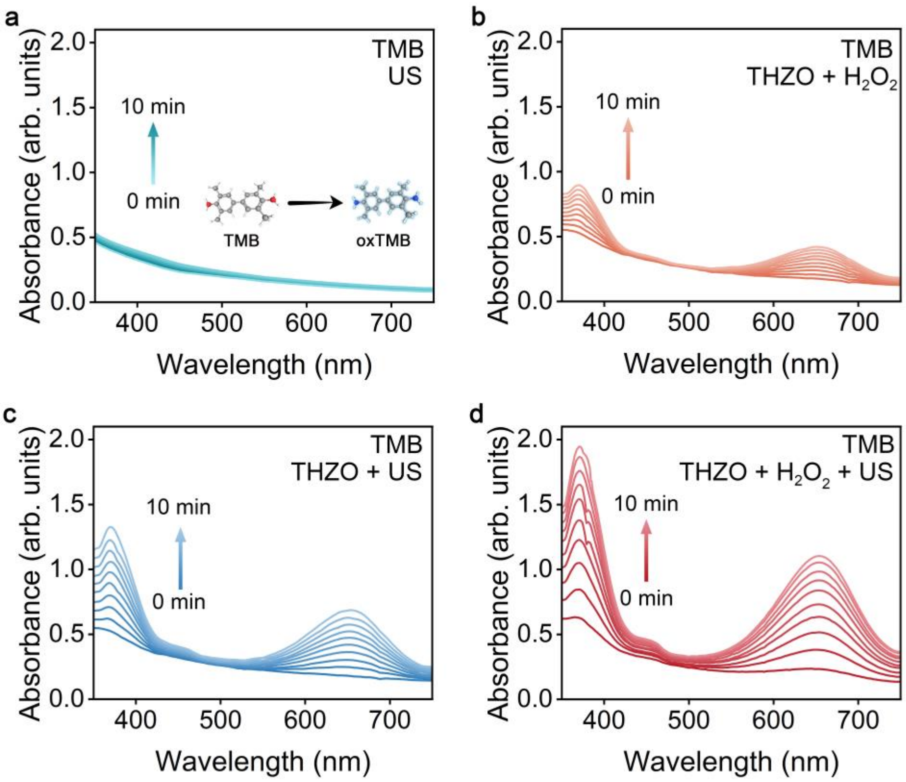

Supplementary Fig. 21. Variation of UV-*vis* absorption peak intensity of THZO NWs with time under different treatment conditions.

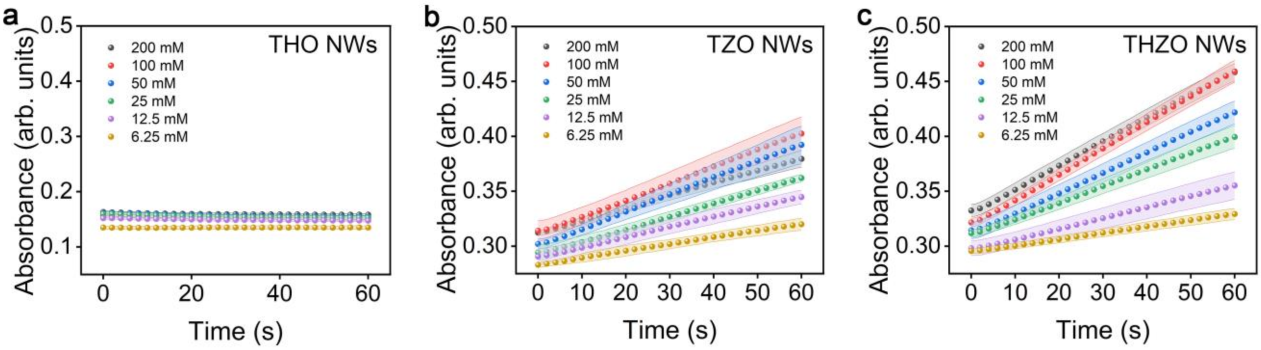

Supplementary Fig. 22. Variation of UV-*vis* absorption peak intensity of **a** TZO NWs, **b** THO NWs, and **c** THZO NWs solutions with time under different concentrations of H_2O_2.

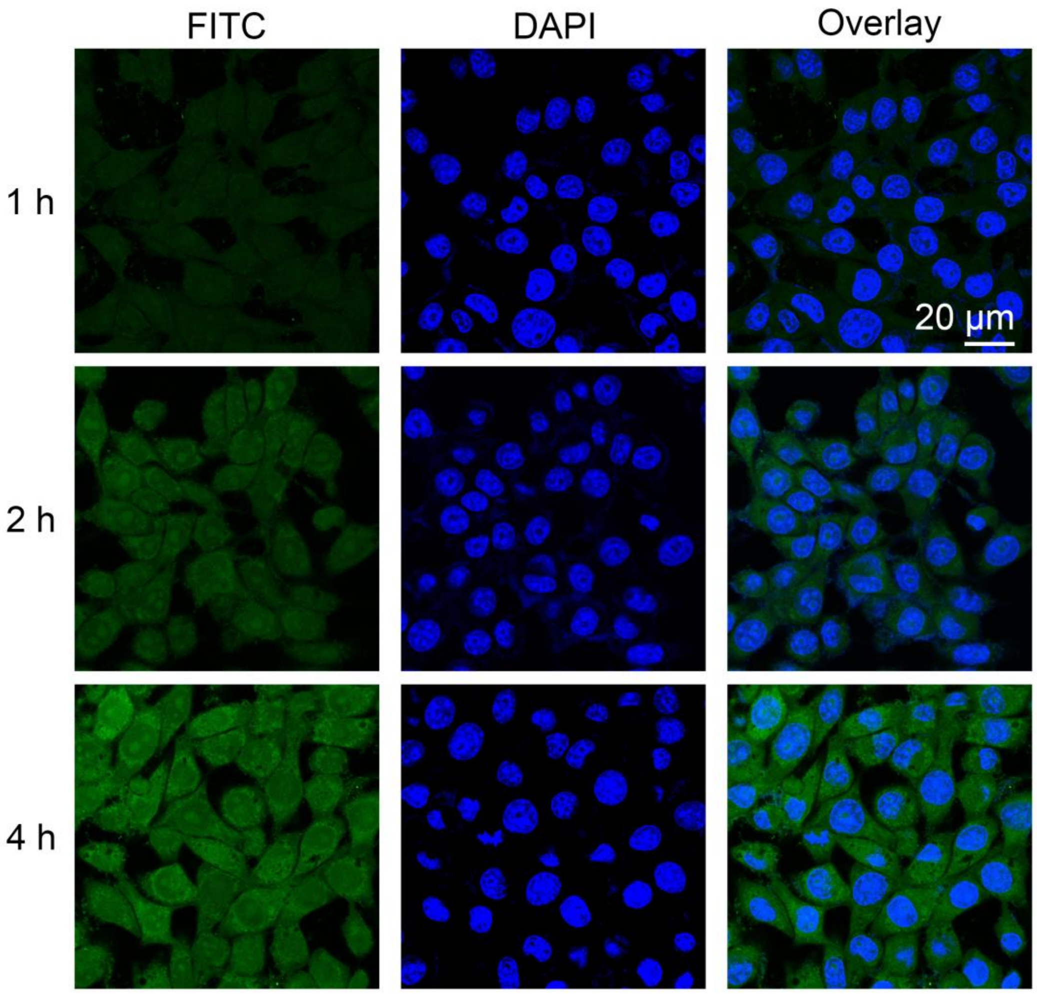

Supplementary Fig. 23. CLSM images of FITC-labeled THZO NWs co-cultured with 4T1 cells after 1, 2, and 4 h.

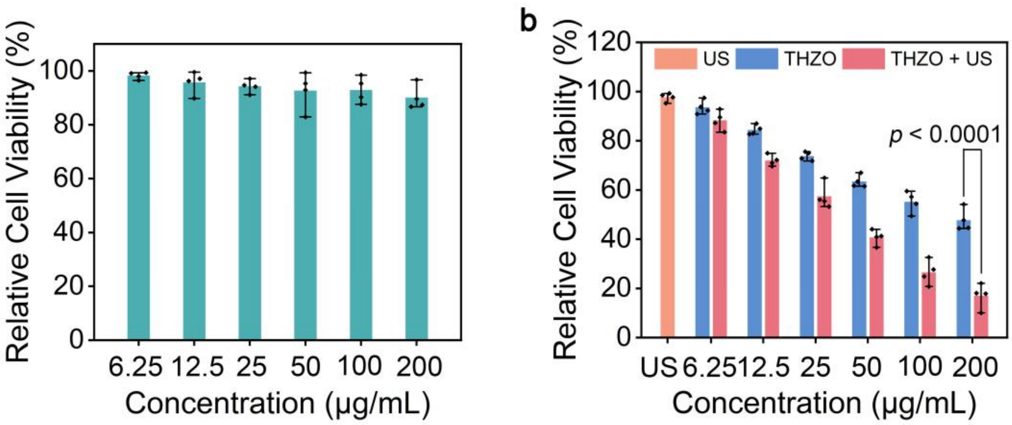

Supplementary Fig. 24. a The relative survival rate of L929 cells incubated with THZO NWs at different concentrations. **b** Relative viabilities of 4T1 cells after being incubated with varied concentrations (200, 100, 50, 25, 12.5, and 6.25 μg mL^{-1}) of THZO NWs and THZO + US (1 MHz, 0.96 W cm^{-2}, 40% duty cycle, 1 min). Data presented as mean ± S.D. (n = 4 biologically independent cell samples).

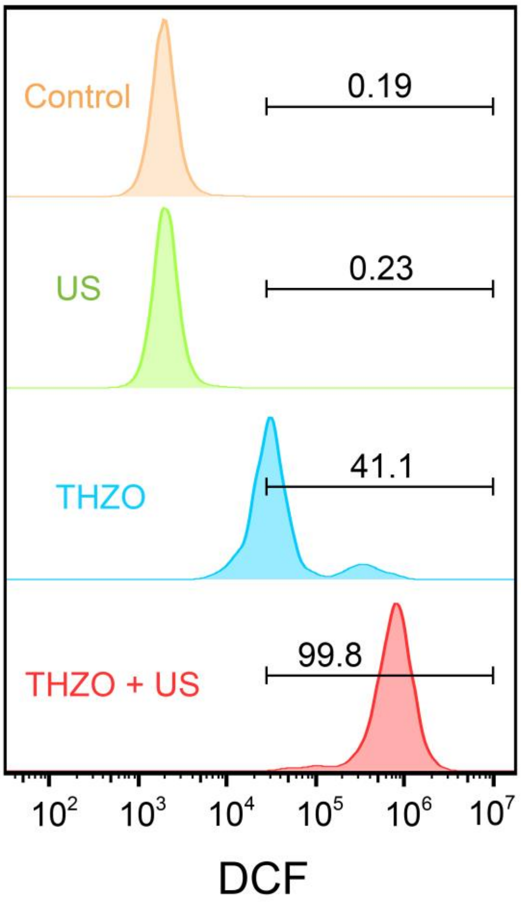

Supplementary Fig. 25. Flow cytometry profiles of ROS levels of different treatments.

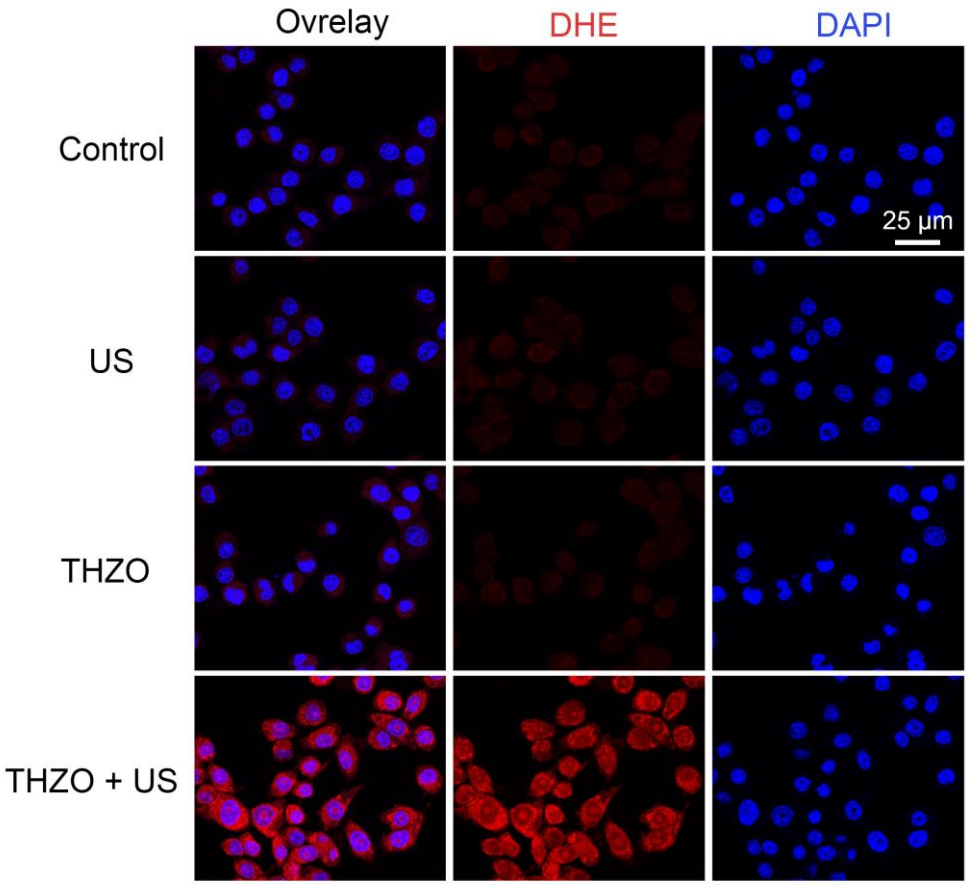

Supplementary Fig. 26. DHE was used as the probe to investigate the fluorescence intensity of $O_2^{\cdot-}$ level in different treatments.

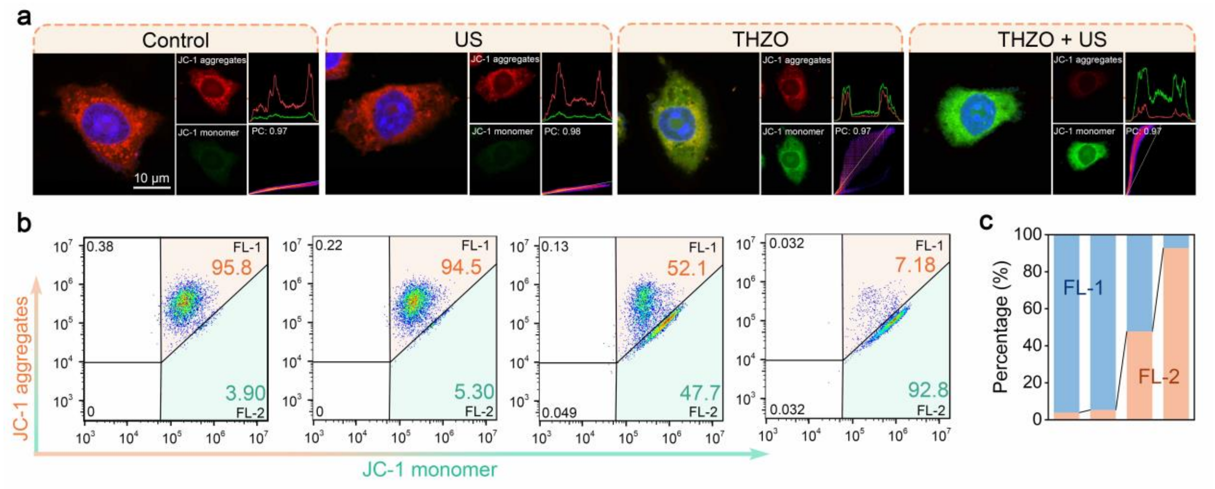

Supplementary Fig. 27. JC-1 staining **a** CLSM images and **b** flow cytometry analysis of 4T1 cells with different treatments. **c** Quantitative analysis of JC-1 staining of 4T1 cells in different treatments.

.

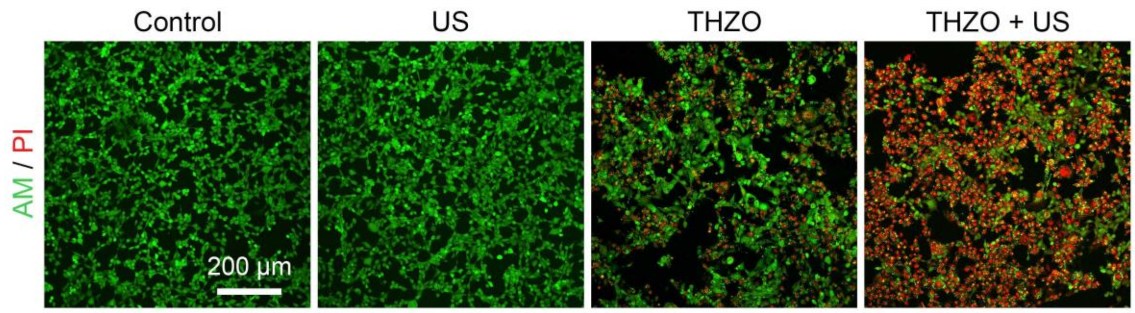

Supplementary Fig. 28. Calcein AM/PI staining of the apoptosis treated with different conditions.

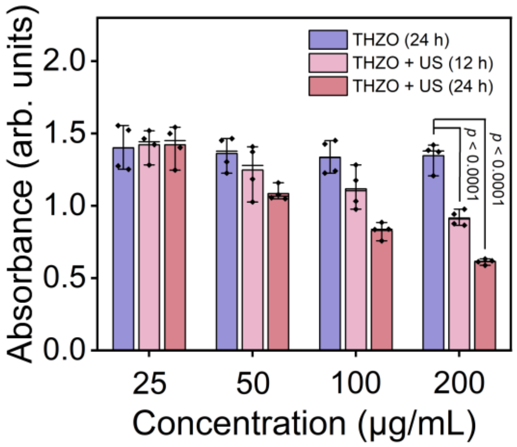

Supplementary Fig. 29. UV-*vis* of 4T1 cells incubated with PTIO for 12 or 24 h under different treatment conditions. Data presented as mean ± S.D. (n = 4 biologically independent cell samples).

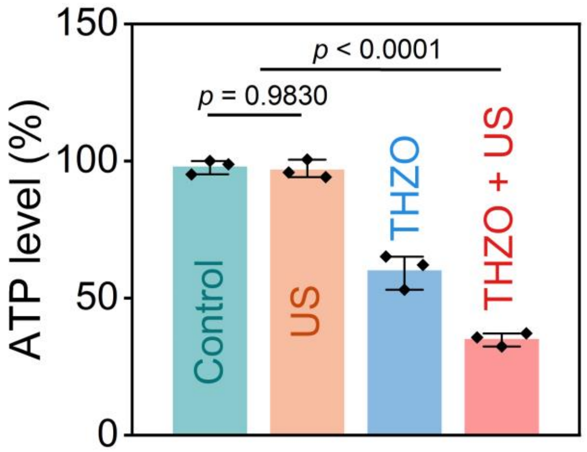

Supplementary Fig. 30. Cellular ATP levels in 4T1 cells subjected to various treatments. Data presented as mean ± S.D. (n = 3 biologically independent cell samples).

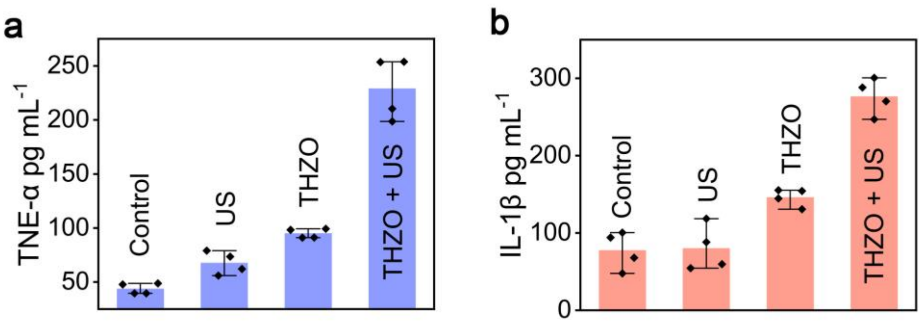

Supplementary Fig. 31. Intracellular **a** TNF-α and **b** IL-1β levels after diverse treatments. Data are expressed as mean ± S.D. (n = 4 biologically independent cell samples).

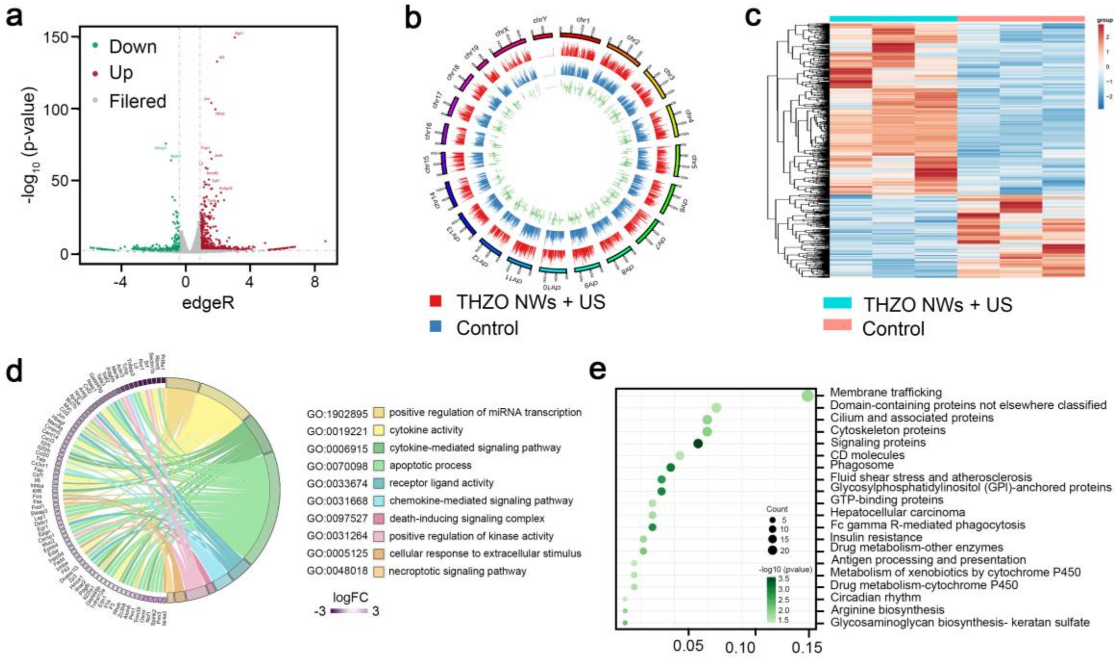

Supplementary Fig. 32. Transcription analysis of the anti-tumor mechanism induced by THZO NWs. **a** Volcano plots and **b** wheel plots of the up-regulated and down-regulated genes of THZO + US compared with the control group. **c** The DEGs clustering graph between THZO + US and the control group. **d** The circle diagram of GO enrichment analysis. **e** KEGG pathway enrichment analysis.

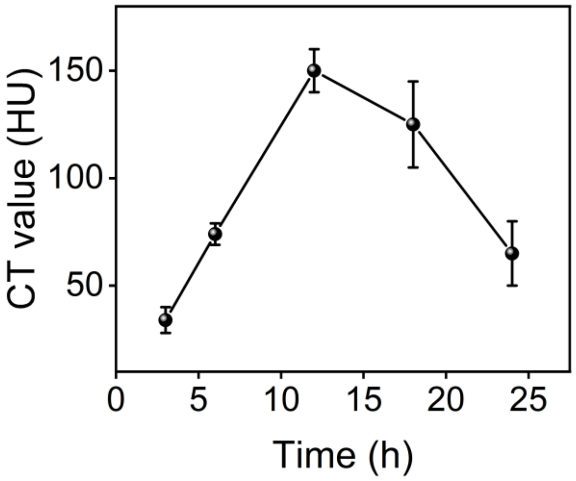

Supplementary Fig. 33. Corresponding CT value variations of the tumor region measured by Image J software. Data are expressed as mean ± S.D. (n = 3 mice).

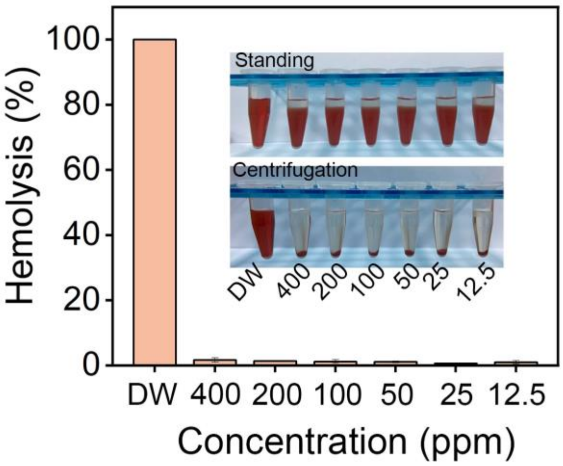

Supplementary Fig. 34. Hemolysis analysis of blood incubated with distilled water (DW, positive) and PBS (negative) at different concentrations of THZO NWs and digital photos 2 h after incubation.

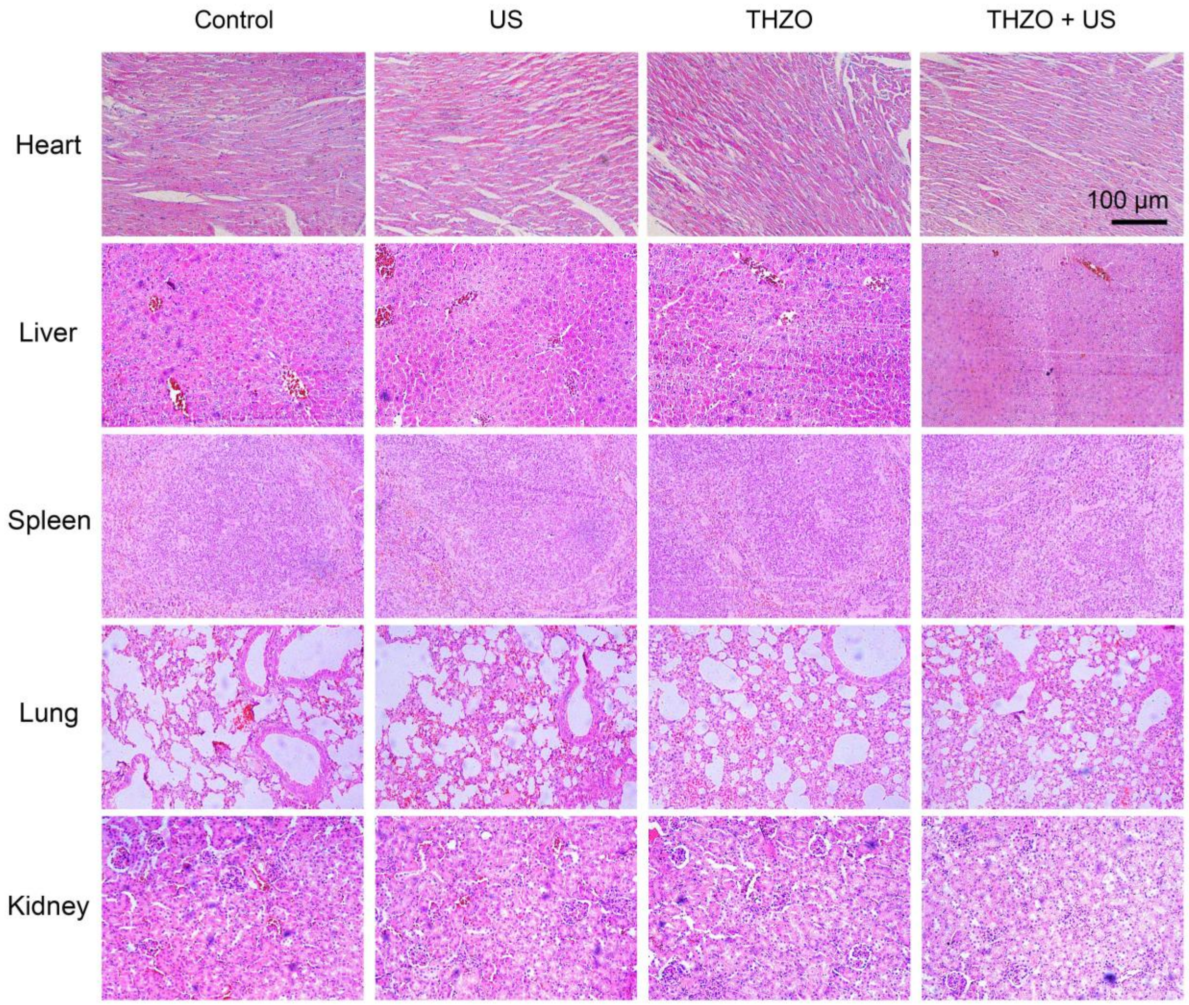

Supplementary Fig. 35. H&E staining images of major organs (heart, liver, spleen, lung, and kidney) of Balb/c mice after intravenous injection of THZO NWs (dose: 10 mg kg^{-1}). Scale bar = 100 µm.

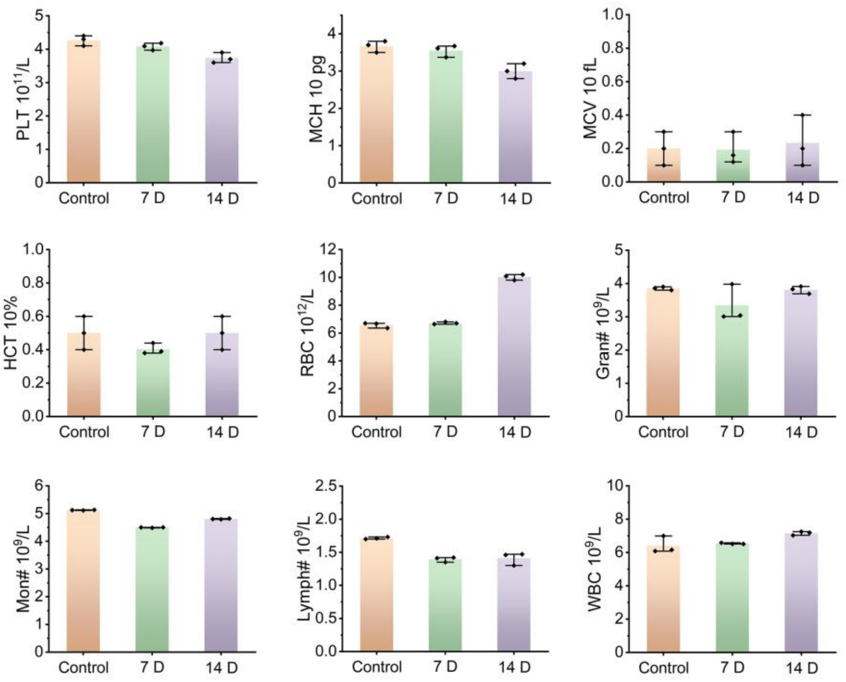

Supplementary Fig. 36. Biochemical blood analysis of the BALB/c mice with the injection of THZO NWs at given time points. Liver and renal function indexes including platelet (PLT), mean corpuscular hemoglobin (MCH), hematokrit (HCT), red blood cells (RBC), neutrophile granulocyte (Gran#), monocyte (Mon#), leukomonocyte (Lymph#), and white blood cell (WBC) were measured. Data are presented as mean ± SD (n = 3 mice).

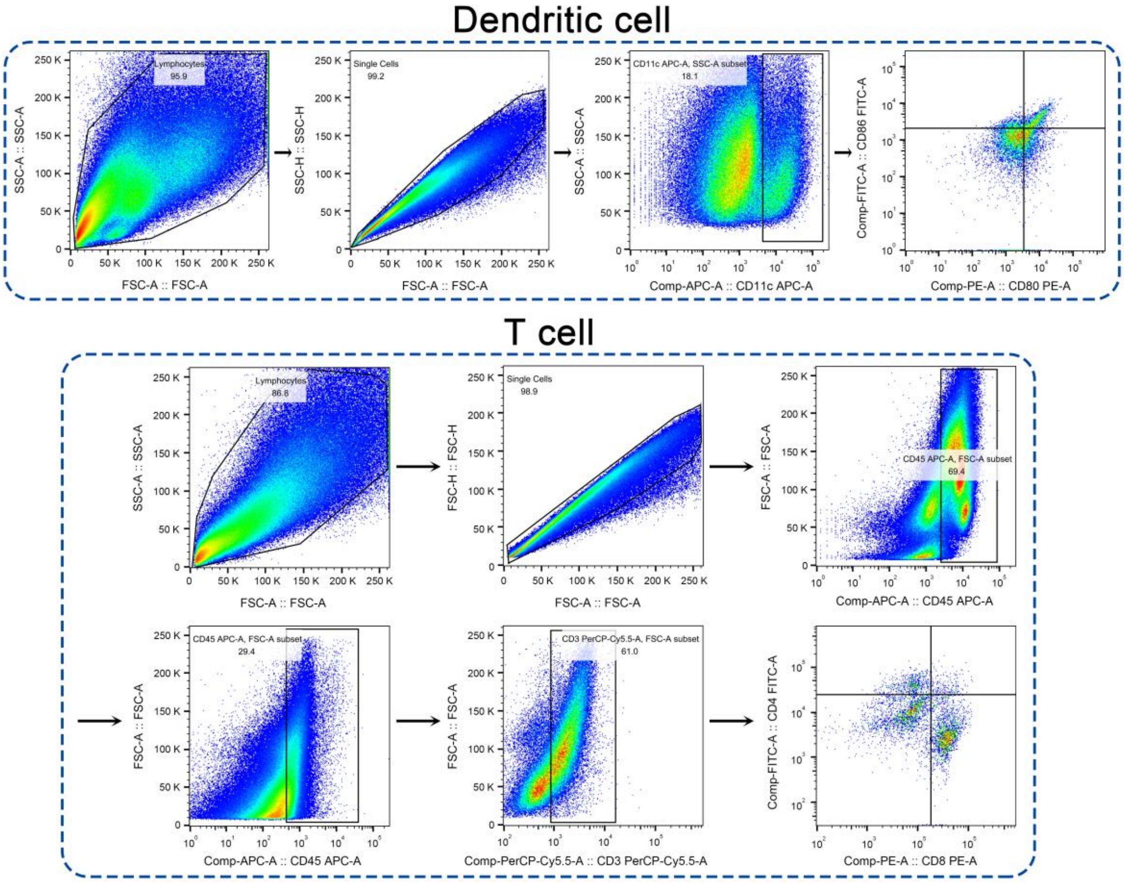

Supplementary Fig. 37. Representative gating strategies for analyzing dendritic cell and T cell.

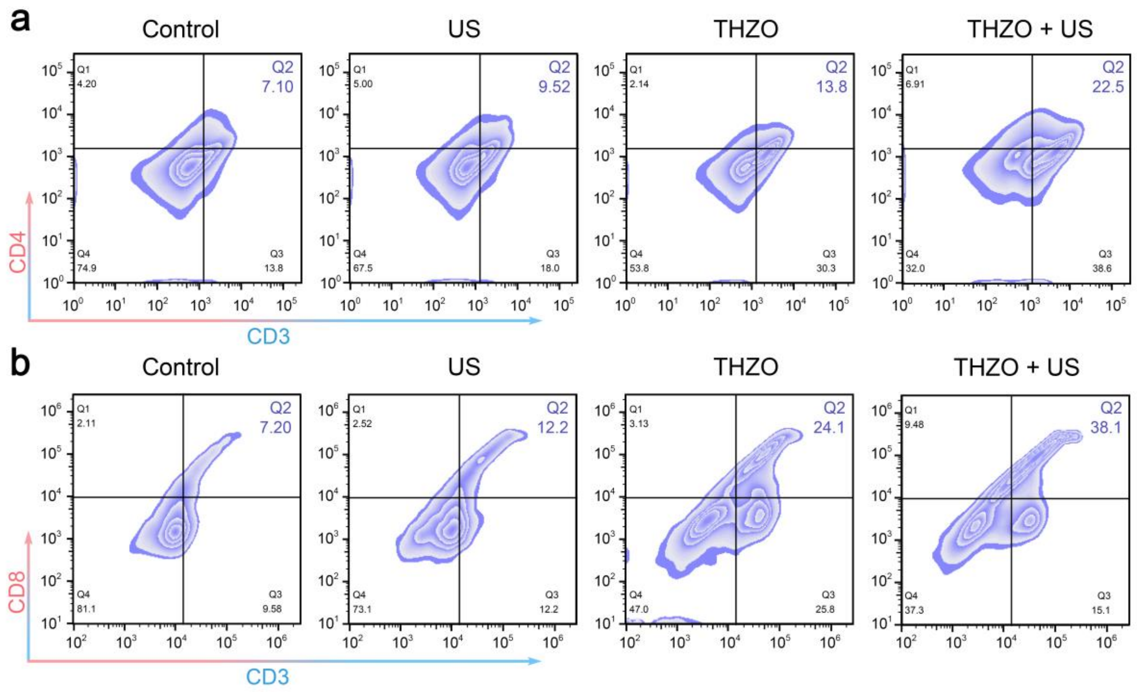

Supplementary Fig. 38. FCM analyses of the percentages of **a** $CD4^+$ and **b** $CD8^+$ T cells in primary tumor of mice after different treatments.

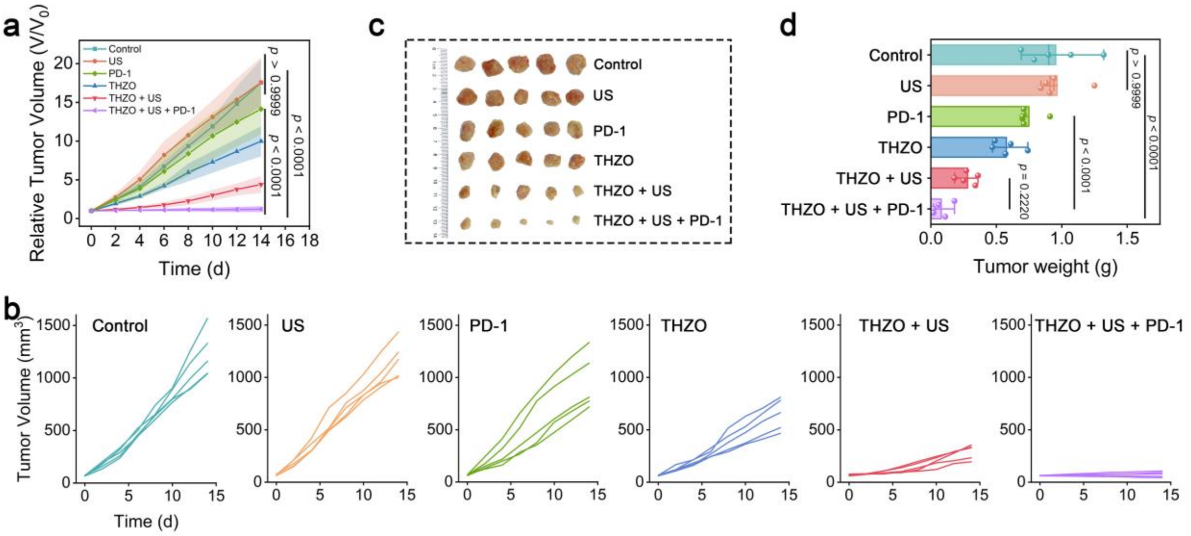

Supplementary Fig. 39. a Relative tumor volume of tumor in 4T1 tumorbearing mice with diverse treatments. Data are expressed as mean ± S.D. (*n* = 5 mice). **b** Relative tumor growth curves from different groups. **c** Typical tumor photographs excised from the mice after different treatments for 14 days. **d** Average weights of tumors harvested from different groups. Statistical analysis was performed *via* unpaired two-tailed Student's t-test. ****$p < 0.0001$, *n. s.*, no significance. Data are expressed as mean ± SD (*n* = 5 mice).

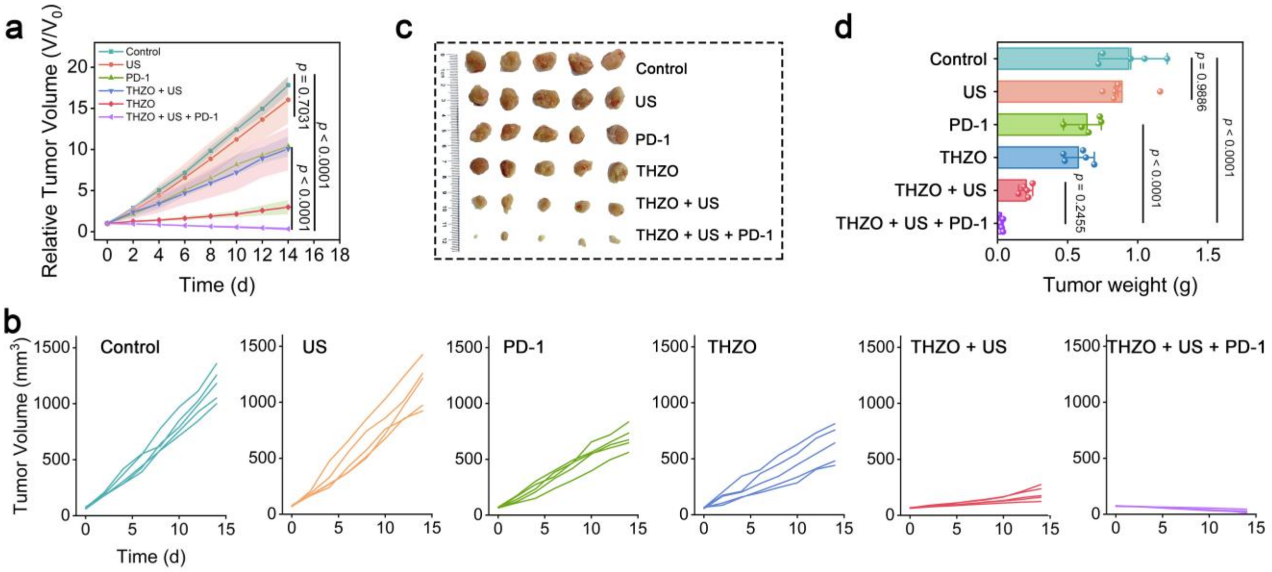

Supplementary Fig. 40. a Tumor volume andmrelative tumor volume of distal tumor in 4T1 tumorbearing mice with diverse treatments. Data are expressed as mean ± S.D. (*n* = 5 mice). **b** Relative tumor growth curves from different groups. **c** Typical tumor photographs excised from the mice after different treatments for 14 days. **d** Average weights of tumors harvested from different groups. Statistical analysis was performed *via* unpaired two-tailed Student’s t-test. ****$p < 0.0001$, *n. s.*, no significance. Data are expressed as mean ± SD (*n* = 5 mice).

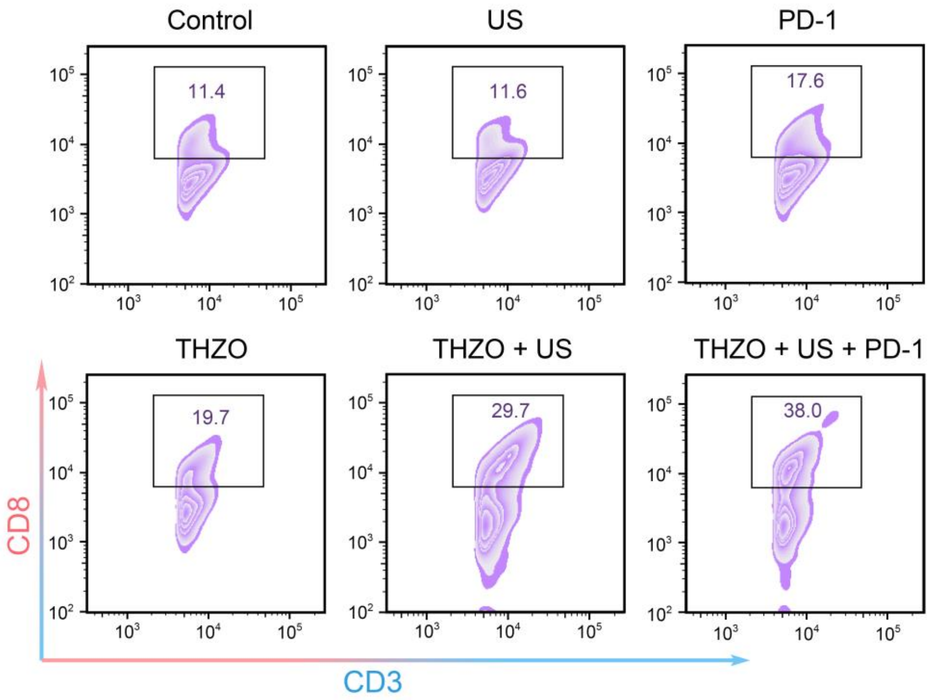

Supplementary Fig. 41. FCM analyses of the percentages of CD8$^+$ T cells in distal tumor of mice after different treatments.

2. Supplementary Discussion

2.1 Structural characterizations

The coexistence of Hf and Zr has a strong influence on the ferroelectric properties.[1, 2] The thermodynamically stable phase of HfO_2 and ZrO_2 has a centrosymmetric crystal structure. Nevertheless, previous experiments have shown that most of its ferroelectric phases (such as the non-centrosymmetric ortho-alternating phases *Pca2₁* and rhomboid phases) are metastable phases, and Zr introduction can stabilize these metastable phases, thereby improving the ferroelectric properties of HfO_2.[3, 4] In addition, the coexistence of Hf and Zr can change the position of metal ions and O atoms, resulting in lattice distortion, which is one of the key factors to improve the ferroelectric properties of HfO_2. In **Scheme 1a**, we present a representative orthogonal HZO NWs model. In this model, the coordination numbers of Hf and Zr clearly show a 1:1 ratio. Further, through the regional element mapping analysis and energy dispersive spectrum (Supplementary Fig. 3), we revealed the fine composition of HZO in depth, confirming that the distribution of Hf, Zr and O elements in the HZO matrix is uniform and reasonable, which provides an essential basis for understanding the microstructure and properties of HZO materials. The valence states of Hf and Zr in the samples were +4 determined by XPS spectroscopy (Supplementary Fig. 4–5). By comparing the high-resolution XPS spectra of HZO NWs with those of HO NWs and ZO NWs, the Hf 4*f* and Zr 3*d* of HZO NWs are red-shifted by 1.25 eV. The important effect of Zr introduction on the electronic structure and lattice distortion of HfO_2 is again confirmed. Furthermore, it can be known from the O 1*s* high-resolution XPS spectral analysis that 530.50 eV corresponds to the C-O covalent bond and 528.85 eV corresponds to the lattice oxygen peak (Supplementary Fig. 6).

During synthesis, HZO NWs are mainly generated in solutions containing octadecene, oleic acid, and oleylamine, and their surface is primarily composed of $-NH_2$ (867 cm^{-1}), C=C (1429 cm^{-1}) and $-CH_2$ (2852 and 2921 cm^{-1}) ligands (Supplementary Fig. 7), which makes it well dispersed in non-polar solvents. However, to apply HZO NWs in organisms, we need to exchange surface ligands while maintaining their polar solvent dispersion. For this purpose, we further functionalized with DSPE-PEG-TPP to obtain TPP-$Hf_{0.5}Zr_{0.5}O_2$ nanowires (THZO NWs), which endows HZO NWs with mitochondrial targeting capability and high biocompatibility. Fourier transform infrared (FTIR) spectra of THZO NWs show benzene ring (1433, 1508, and 1596 cm^{-1}) and single substitution characteristic double peaks (697 cm^{-1} and 743 cm^{-1}). Meanwhile, the Zeta potential of the HZO NWs surface shifts from the original −20.61 mV to +15.62 mV, again demonstrating

the exchange of surface ligands (Supplementary Fig. 8). After such surface modification, the THZO NWs can be stably dispersed in water, which not only maintains their ultrafine-linear morphology but also achieves a homogeneous dispersion state, laying a solid foundation for subsequent biological applications (Supplementary Fig. 9). Through *in vitro* rheological experiments, it was detected that within the shear rate range of 1–100 s^{-1}, the NWs dispersion exhibited non-Newtonian fluid characteristics, confirming its dispersibility in the actual blood flow environment. Meanwhile, when THZO NWs was incubated in distilled water, normal saline and simulated body fluids (PBS containing 10% FBS) for 24 h, no obvious agglomeration phenomenon was observed in the TEM images. It is proved that THZO NWs can maintain good dispersion in the complex ionic environment of the human body.

After being placed in a non-polar solution for a period of time (10 h), a gel-like form is formed, confirming the polymer-like flexibility of HZO NWs (Supplementary Fig. 10). As shown in Supplementary Fig. 11, the corresponding tensile strain-tensile stress curve was obtained through the tensile property test. After adding THZO NWs to the hydrogel, the mechanical properties were improved to a certain extent. The tensile capacity of THZO NWs (1299%) is higher than that of the original hydrogel (1030%), and the tensile strength of THZO NWs (993 Pa) is slightly higher than that of the original hydrogel (844 Pa). However, the tensile capacity and strength of the composite hydrogel with the same content of THZO NPs are inferior to those of THZO NWs. The variation trends of Young's modulus and toughness curves corresponding to the original hydrogel, the composite hydrogel of THZO NPs and THZO NWs, are the same as those of the stress-strain curve. THZO NWs has the highest Young's modulus (38.29 KPa) and the highest toughness (0.72 MJ m^{-3}).

2.2 ROS generation property

To investigate the role of the main reactive oxygen species (ROS) sincerely, we conducted sacrificial agent experiments (Supplementary Fig. 15). The results revealed that the degradation rate of RhB was significantly enhanced by adding the sacrificial agent p-benzoquinone (p-BQ, used for scavenging $O_2^{\cdot-}$). At the same time, tert-butanol (TBA), a sacrifice agent used to remove ·OH, has a specific inhibitory effect on the degradation of RhB, which fully indicates that ·OH plays a crucial role in the degradation process and is the main component of ROS. Surprisingly, however, the degradation of RhB was inhibited entirely when 2,2,6,6-

tetramethylpiperidine (TEMP, a sacrificial agent for $^1O_2$) was added. This suggests that $^1O_2$ is produced by reacting $O_2\cdot^-$ and ·OH.

Next, to gain a deeper understanding of the POD-like activity of THZO NWs, we selected 3,3',5,5'-tetramethylbenzidine (TMB) and H_2O_2 as the reaction substrates and conducted steady-state catalytic kinetic comparison experiments for the three materials in phosphate-buffered saline (PBS, pH = 5.5). Supplementary Fig. 17 displays the trend of the absorbance over time as the colorless TMB is oxidized to blue oxidized TMB (oxTMB). The absorbance of THO NWs remains almost unchanged, while that of TZO NWs and THZO NWs increases significantly. We accurately determined the initial rate (v_0) of ·OH production by applying the Beer-Lambert law. Further research revealed that the reaction rate exhibits a notable linear relationship with the concentration of H_2O_2, a finding that aligns with the description of the Michaelis-Menten equation (**Fig. 3k**). In this equation, [S] represents the concentration of the substrate. To obtain more information about the kinetics of the enzymatic reaction, we further plotted the Lineweaver-Burk diagram (**Fig. 3i**). We derived the maximum reaction velocity (V_{max}) and Michaelis-Menten constant (K_m). The V_{max} and K_m of THZO NWs were 6.38×10^{-8} M S^{-1} and 18.95 mM, respectively, at room temperature. This result suggests that THZO NWs have an excellent affinity for H_2O_2, which enables an efficient enzymatic reaction.

$$v_0 = \frac{V_{max} \cdot [S]}{K_m + [S]} \quad (1)$$

$$\frac{1}{v_0} = \frac{K_m}{V_{max}} \cdot \frac{1}{[S]} + \frac{1}{V_{max}} \quad (2)$$

In summary, the efficient and stable CAT-like and POD-like enzymatic activities exhibited by THZO NWs are entirely attributed to the effective decomposition of H_2O_2 by their internal ZrO_2. This discovery offers new insights into the application of inorganic nanomaterials in the field of enzymatic therapy. Furthermore, the potential of THZO NWs as piezoelectric materials has been fully validated, demonstrating their capability for synergistic piezoelectric and enzymatic therapy.

2.3 *In vitro* cytotoxicity assessment

In vitro cytotoxicity against normal cells demonstrated negligible cytotoxicity of THZO NWs against L929 fibroblasts (Supplementary Fig. 19a). Subsequently, we further evaluated the *in vitro* antitumor efficacy of THZO NWs by the methyl thiazolyl tetrazolium (MTT) assay. As shown in Supplementary Fig. 19b, US irradiation had a negligible inhibitory effect on cell viability compared to the control. In contrast, the survival

rate significantly decreased with increasing concentration after co-incubation with THZO NWs, attributed to the therapeutic effect of ·OH produced by POD enzyme activity. Significantly, the viability of cells treated with THZO NWs decreased dramatically to 19.3% under US irradiation, confirming the effective therapeutic effect of THZO NWs.

2.4 Mitochondrial function

Then, we applied JC-1 dye as an indicator of mitochondrial function to detect mitochondrial damage after THZO NWs treatment. Under normal physiological state, mitochondria have high negative electronegativity, JC-1 enters into mitochondria to exist as multimers with red solid fluorescence, and during apoptosis, mitochondrial depolarization arises, negative electronegativity decreases, and JC-1 exists in the cytoplasm as monomers with enhanced green fluorescence. The decrease in the red/green fluorescence intensity ratio can judge the extent of mitochondrial damage. As shown in Supplementary Fig. 22a, cells in the untreated group showed red solid fluorescence, indicating that the change in mitochondrial membrane potential was insignificant. Cells treated with US irradiation alone were in good condition, with intense red and weak green fluorescence. However, cells treated with THZO showed a significant decrease in red fluorescence intensity, reflecting the loss of mitochondrial membrane potential. Especially in the THZO + US group, a large amount of ROS was generated due to the combined effect of enzyme catalysis and piezoelectric catalysis, and these ROS led to oxidative damage of the mitochondrial membrane, which in turn caused the loss of mitochondrial membrane potential and ultimately induced cell apoptosis. After US irradiation, the red fluorescence intensity of the cells decreased significantly, indicating the most significant loss of mitochondrial membrane potential. At the same time, we detected apoptosis using flow cytometry, with green fluorescence detected through the FL-1 channel and red fluorescence detected through the FL-2 channel (Supplementary Fig. 22b–c). After treating of the THZO group and THZO + US group, the FL-1 channel gradually increased and reached 92.8%. The results fully demonstrated that TPP-induced mitochondrial targeting combined with the massive generation of ROS effectively disrupted mitochondrial function.

3. Supplementary References

1. Park, MH., *et al.* Ferroelectricity and antiferroelectricity of doped thin HfO_2-based films. *Adv. Mater.* **27**, 1811–1831 (2015).

2. Song, T., *et al.* Stabilization of the ferroelectric phase in epitaxial $Hf_{1-x}Zr_xO_2$ enabling coexistence of ferroelectric and enhanced piezoelectric properties. *ACS Appl. Electron. Mater.* **3**, 2106–2113 (2021).

3. Zhu, X., *et al.* Impact of proton radiation on Zr-doped HfO_2-based ferroelectric memory. *J. Alloys Compd.* **1002**, 175372 (2024).

4. Xi, Y., *et al.* Effects of oxygen flow during fabrication by magnetron sputtering on structure and performance of Zr-doped HfO_2 thin films. *Materials* **16**, 5559 (2023).
