# Supplementary material for: Constructing flexible sub-nanometer ferroelectric catalyst to overcome heterocatalytic kinetic barriers for enhanced catalytic and immuno-therapy
Source: Nat Commun. 2025 Dec 16;17:404. doi: 10.1038/s41467-025-67097-6 (PMC12796250; doi:10.1038/s41467-025-67097-6)
Supplement: Supplementary file 2 — Description of Additional Supplementary Files [file 41467_2025_67097_MOESM2_ESM.pdf]

Title: Supplementary Movie

Description: Supplementary Movie is designed to demonstrate the molecular dynamics simulation process and to show the structural strain caused by vibrations.

Title: Supplementary Data

Description: Supplementary Data is the density functional theory model
